# How Do We Meet the Supportive Care and Information Needs of Those Living With and Beyond Bladder Cancer?

**DOI:** 10.3389/fonc.2020.00465

**Published:** 2020-04-08

**Authors:** Sara Jane MacLennan, Steven MacLennan

**Affiliations:** Academic Urology Unit, University of Aberdeen, Aberdeen, United Kingdom

**Keywords:** bladder cancer, supportive care, information, unmet needs, quality of life

## Abstract

This perspective paper presents the case for adopting a new approach to the design and delivery of supportive care for those with bladder cancer. It is our assertion that the design and delivery of supportive care for those diagnosed with bladder cancer needs to (1) build on existing research and available tools and (2) address current limitations due to lack of use of said tools, lack of understanding of research and needs, lack of a shared language, and method of assessment and evaluation. This, we argue, can be achieved through a network-based approach (1) focussed on the structure, process, and outcome of supportive care.

## Introduction

There is growing agreement across all tumor types that we need to treat the disease-condition and to meet the information and supportive care needs of those living with and beyond cancer; to support individuals to have a life lived well (2). A diagnosis of bladder cancer (non-muscle invasive and muscle-invasive) is a stressful life event with numerous supportive care needs that continue beyond initial treatment and yet there is a lack of research around informational and supportive care needs of this group (3). Quality of life and unmet needs have not been well-researched across the whole illness trajectory which in part is due to a lack of high quality measurement instruments that are consistently used. This perspective paper acknowledges the need for better information and supportive care across the cancer journey and outlines a vision for how this might be achieved.

Over the past 5 years, there have been a handful of studies that have attempted to describe or map the information and supportive care needs of those diagnosed with bladder cancer from the perspective of the individual (3–8). There are few studies which have looked at the understanding of these needs from the other main stakeholder groups (health care professionals (e.g., urologists, oncologists, cancer nurse specialists), researchers and non-profit organizations) in the design and delivery of care.

The majority of studies have focused on unmet needs around aspects of (1) the experience of bladder cancer and treatment (such as living with a urostomy or sexual function following cystectomy), (2) quality of life and domains of functioning (such as cognitive, social, sexual, and emotional) and (3) experience of and satisfaction with care (support, information, continuity, burden, and inconvenience). A small number of these have attempted to map these needs across the cancer journey. Some studies conclude that reported needs appear to be being met through current care systems and delivery; others that there remain large gaps in both our understanding and in closing the gap between research and the design and delivery of care. It is difficult to draw substantive conclusions across these studies due to the heterogeneity of the research; studies around mapping the unmet needs of those diagnosed with bladder cancer have measured slightly different dimensions in different ways for different groups of individuals [different types of bladder cancer: non-muscle invasive (NMIBC) and muscle-invasive (MIBC)] at different stages in their cancer journey. Examples of key studies are given below and are organized by (1) NMIBC, (2) MIBC, and (3) changing needs across the cancer journey. This is not intended to be a systematic review of the literature but is provided to allow the reader insight in to the current knowledge landscape.

Focusing on NMIBC, Rutherford et al. (9) developed a framework to describe the experience of living with and beyond a diagnosis of NMIBC. This included three key domains—(1) the disease-condition (symptoms and treatment including blood in urine; frequency, urgency, incontinence; pain when urinating, pelvic pain; nausea, vomiting, constipation, diarrhea; fatigue, loss of sleep; infection, fever; skin rashes), (2) three dimensions of functioning (cognitive, sexual functioning, emotional functioning) and (3) experiences and satisfaction with care (including support, information, continuity, burden, and inconvenience.

Focusing on MIBC, Mohamed et al. (8) explored informational (information support) and supportive care needs (medical, psychological, and emotional support) across the illness trajectory. They found that individuals reported unmet needs across five key domains—(1) health system and information needs, (2) patient care and support, (3) physical/daily living, (4) psychological well-being, and (5) sexuality. Mohamed et al. (5) then conducted a further investigation of this data and found that the unmet needs of those living with MIBC vary by age, sex, and treatment choice. They make the argument that assessment and intervention needs to be tailored to these specific needs. An argument supported by Bhanvadia (7) who also found that needs differ along racial, gender, and socio-economic groups. They too highlighted the importance of long-term support and survivorship resources and for tailored models that address quality of life and supportive care needs across the patient journey.

Paterson et al. (5) systematically reviewed and summarized the literature on supportive care needs of those with MIBC. Their paper characterizes supportive care needs in nine domains: (1) patient-clinician communication, (2) daily living needs, (3) health system/information needs, (4) practical needs, (5) family-related needs, (6) social needs, (7) psychological needs, (8) physical needs, and (9) intimacy needs. They reported that individuals with MIBC expressed high unmet needs at diagnosis and these continued beyond primary treatment. The paper acknowledged that understanding of how needs mapped across the cancer journey is still needed.

Focusing on changing needs across the cancer journey, Edmonson et al. (7) and Chung et al. (3) assessed information and supportive care needs across the illness trajectory. Both studies highlighted changes in quality of life and supportive care needs over time and argued for the need for further research (3) and better measurement of key outcomes across the cancer journey (7).

Edmonson et al. (7), through an in-depth review of the qualitative literature, mapped the lived experience and needs of those with NMIBC and MIBC on to the individual's cancer journey at diagnosis, during acute care and treatment, post-treatment, and beyond (which they name as “the new normal”). This is the first in-depth systematic review of the qualitative evidence in this area. This paper allows greater insight in to the lived experience of bladder cancer and changes in supportive care needs over time. Edmonson et al. (7) clearly highlight the need for further and better quality research in this neglected area.

Chung et al. (3) looked at quality of life, informational and supportive care needs of individuals with NMIBC and MIBC across the illness trajectory. The key supportive care needs reported were about (1) sex life; (2) decisions about life in uncertainty; (3) coping with others not acknowledging impact of cancer; (4) coping with expectations of individual as cancer survivor; (5) coping with change to belief that nothing bad will happen in life; (6) developing new relationships after cancer; (7) understanding financial entitlements; (8) accessible hospital parking; (9) impact on relationship with partner; and (10) life/travel insurance. They described these in terms of existential care needs. Most of the reported information needs were in the medical domain (knowledge of cancer, treatment options, side effects, subsequent post-treatment tests). Encouragingly, they found that individuals reported most of the identified supportive care needs had been met.

Despite these studies, gaps still exist within our knowledge of how needs change over time. Less is known about how we translate research in to practice and best meet these needs at different points across the cancer pathway (3, 7), particularly the further we move from primary treatment. We can all identify strong examples of existing good clinical practice but these are grounded in the expertise of the different teams in the different geographical locations in meeting the supportive care and information needs of their patient groups. The question becomes how do we ensure that this is the typical experience for all those diagnosed with bladder cancer within the UK, and globally?

In this perspective paper, we argue that there is a need to think more broadly in terms of the involvement and role of stakeholders in understanding supportive care needs and changing behavior. This would take the form of a multi-stakeholder approach that (1) builds a community of expertise, (2) is grounded in a pathway perspective and (3) allows the individual to be an active participant in the design and delivery of supportive care and appropriate information.

**Figure 1:**
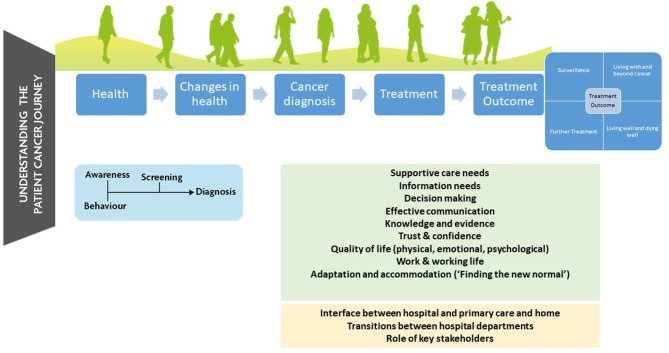
Mapping the supportive care and information needs of those diagnosed with cancer.

The current view of the authors is based on an extensive programme of work over the past 10 years from the University of Aberdeen to better understand the information and supportive care needs of those diagnosed with all forms of urological cancer (including bladder cancer) across the cancer journey. The focus of this has been on structure, process (key stakeholder behavior and interaction) and outcome (measurement and definition) of cancer care (10). This has included co-design work looking to address supportive care and information needs of those diagnosed with urological cancer (11), understanding how best to include the individual's voice in the design and delivery of care, and the language and measurement of care and outcomes e.g., clinical guidelines and core outcome sets [For more detail on individual projects see (1, 12–18)] (see Figure 1).

This has culminated in an understanding that the design and delivery of supportive care and timely and appropriate information is necessarily more complex than a simple allocation of responsibility for providing this care to one stakeholder group or role or individual. This responsibility has to be shared across stakeholder groups across the time line of the person's journey from diagnosis to decision making to treatment to follow-up and continuing forward. Essentially, a network-based approach (1) to the design and delivery of care grounded in understanding *the process* of these interactions between stakeholder groups.

A network-based approach requires a number of things to be successful. First, it requires the main stakeholder groups to understand each other's roles, priorities, expertise, level of knowledge and behavioral drivers. This would be achieved through characterizing knowledge, attitudes, and the determinants of the key behaviors [e.g., using the theoretical domains framework (19)]. Second, it requires that each stakeholder group is confident in their own knowledge and the accepted boundaries of their own competence. They also need to be confident in the knowledge and competence of those who they can signpost to within this process. Third, it requires better, more informed communication between individuals as well as within and between stakeholder groups which is supported by appropriate structures and a reliable method to measure this (e.g., are treatment and care achieving the outcomes that matter most to the key stakeholder groups?). Step two and three build on step one and require the identification of behavior change techniques to change clinical practice and improve uptake of evidence in to practice [e.g., COM-B systems of behavior change (20)].

If we accept the need for a network-based approach (*process*), this also has to be supported by education and training. First, education is required in terms of the nature and current availability of reliable and useful information about bladder cancer and treatment and how best to tailor this to the individual's diagnosis and cancer journey to support shared-decision making. Second, training and informed discussion are required in terms of appropriate ways of delivering that information and when. Third, training and informed discussion are required to support the development of local networks and role profiles and an appropriate structure to achieve this. Such education and training could be provided by one of the major independent actors in this area, for example, Fight Bladder Cancer, Macmillan Cancer Support.

The need for *appropriate structures* of care is important in achieving the aim of better supportive care and information. There is an argument for building on existing structures to include behavior and the interactions between the main stakeholder groups. This also needs to keep pace with developments in diagnosis and treatment (e.g., personalized medicine, big data and prognostic and predictive biomarkers). This could take many forms but would have three key functions: (1) to collect information from the individual, (2) to process in real-time and to deliver appropriate and evidence-based information back to the individual and (3) act as a record of these “conversations” for future reference and for review.

Examples of existing structures include the recognition of information and supportive care needs as a priority within clinical practice guidelines for bladder cancer [e.g., (21)] and the availability of tools and systems to (1) assess healthcare needs, (2) provide access to information and resources, and (3) to inform recommendations for healthcare teams and for individuals (care plan and treatment summary) [e.g., the National Cancer Survivorship Initiative and Macmillan's Recovery Package and the online Holistic Needs Assessment tool (eHNA) through mycareplan (2)].

In an ideal world, every individual would have access to a platform that would collect and collate PROMs in real-time and flag information and supportive care needs to the healthcare team and the individual across the cancer journey. This would support shared decision-making and person-centered care. This would also act as a platform to capture the information and supportive care delivered and to map communication with the capacity to “learn” over time. Existence of such a record would also encourage reflection on advice given, support consistency in that advice and facilitate better communication. This record would also be a summary of the different stakeholder roles in the process.

The important point to highlight about *structure* is that it needs to be informed by *process* and *outcome*. The development of new online and evidence-based tools to provide information such as those flagged are necessary but not sufficient to fully address the problem. In addition to the development of such evidence-based tools, we need to understand the process in which these are used and useful and the outcomes that are being measured and reported.

We have outlined our proposal for two essential elements in the design and delivery of supportive care and information (*process* and *appropriate structures*). The final element in this is *outcomes*. A move toward the design and delivery of evidence-based supportive care needs to be matched by better measurement and the inclusion of core outcomes as trial end points and in big data. When we try to overview all the evidence that has been reported for the available treatments for bladder cancer, we often find that it is difficult to compare, contrast and summarize it. A main reason for this is heterogeneity in outcome reporting and definitions. That is, many studies comparing the same interventions in the same patient populations use different outcomes or define the same outcomes in different ways. This in turn makes it difficult to make recommendations for treatment in clinical guidelines and it hampers decision making for clinicians and patients. A solution to this is to create core outcome sets (COS). A COS is an agreed standardized collection of outcomes which should be measured and reported, as a minimum, in all trials for a specific clinical area (22). These may be extended to data collection in routine practice, as proposed by ICHOM (23).

A focus of COS development is that patients should be involved as key stakeholders in prioritizing which outcomes are most important to them and thereby ought to be measured in future trials or day to day clinical practice. Work is ongoing to develop a COS for the various stages of bladder cancer (http://www.comet-initiative.org/studies/details/1135). This will assess to what extent currently available measures are fit for purpose and if new tools need to be developed which better reflect patient's concerns. In future, when the most important outcomes are consistently measured in the same ways across trials, it will be easier facilitate treatment decision-making and monitor patient's outcomes across the cancer journey so that timely intervention may be prompted. This links back to the processes and systems we have already discussed.

It is our firm belief that the design and delivery of supportive care for those diagnosed with bladder cancer needs to (1) build on existing research and available tools (e.g., celebrate success and not reinvent the wheel and (2) address current limitations due to lack of use of existing tools, lack of understanding of existing research and examples of best practice, lack of a shared language, and method of assessment and evaluation. This can be achieved through a network-based approach focussed on the structure, process and outcome of supportive care.

## Discussion

In conclusion, there is a very real need to continue progress and build on successes in this area to better support those living with bladder cancer. The intention behind this paper is to act as a nudge to researchers and healthcare professionals working within this area to commit to action and drive change. We can achieve this by working to deliver research in to the design and delivery of information and supportive care for bladder cancer that is grounded in a network-based approach. We need to be able to bring together research identifying the unmet needs of those diagnosed with NMIBC and MIBC and core outcomes, current clinical practice and excellent supportive care and strong information resources championed by non-profits orgs such as Fight Bladder Cancer. This should also inform policy and healthcare planning, the commissioning of resources and clinical practice guidelines. As we have proposed during this brief paper, there is a clear role for the key stakeholder groups (individuals living with and beyond bladder cancer, health care professionals (urologists, oncologists, cancer nurse specialists, primary care), researchers and non-profit organizations) to come together to innovate and communicate. The solution lies in working together. What is needed now is action.

## Author Contributions

SJM and SM contributed substantively to the development of the perspective described within the manuscript and the preparation of the manuscript.

### Conflict of Interest

The authors declare that the research was conducted in the absence of any commercial or financial relationships that could be construed as a potential conflict of interest.
